# Presenting a food in multiple smaller units increases expected satiety

**DOI:** 10.1016/j.appet.2017.07.024

**Published:** 2017-11-01

**Authors:** Rose E. Oldham-Cooper, Laura L. Wilkinson, Charlotte A. Hardman, Peter J. Rogers, Jeffrey M. Brunstrom

**Affiliations:** School of Experimental Psychology, University of Bristol, 12a Priory Road, Bristol, UK

**Keywords:** Expected satiety, Segmentation, Perceived volume, Portion size, Energy intake

## Abstract

Presentation of the same amount of a food in multiple smaller units (‘segmentation’) has been shown to reduce food intake and increase estimates of the amount of food consumed. However, this effect has been demonstrated for *ad libitum* food intake only. In the majority of cases, meals are not consumed *ad libitum*, but are pre-selected and consumed in their entirety, Expected satiety (ES; the anticipated capacity of a portion of food to relieve hunger between meals) is an excellent predictor of portion size selection. This study tested the hypothesis that segmentation increases ES. It was also hypothesised that perceived volume (PV) may account for the relationship between segmentation and ES. Sixty-eight participants made computer-based ES and PV judgments for equicaloric portions of three test foods (salted peanuts, spaghetti Bolognese, and chicken tikka masala), which were presented in either a single unit or as multiple smaller units (three or six units). Results revealed a consistent effect of segmentation on ES - foods presented in multiple smaller units were expected to deliver significantly greater satiety than when presented in a single unit (*p* < 0.005). Furthermore, results indicated that the effect of segmentation on ES was attributable to an increase in PV. ES plays an important role in determining the portion sizes that people select. Therefore, awareness of the effect of segmentation on ES may help to inform the design of foods that confer benefits for healthy weight maintenance.

## 1 Introduction

A number of studies have demonstrated that presenting a food in multiple small units reduces subsequent food intake and increases estimates of the amount consumed (Marchiori et al., 2011, Marchiori et al., 2012, Wadhera et al., 2012, Weijzen et al., 2008). In one study, Chang et al. (2012) served rice in either an amorphous mass or in smaller units (rice balls). Participants consumed less rice when it was served in smaller units relative to an amorphous mass (323 kcal vs. 412 kcal respectively, a 28% difference). In another study, coloured potato chips inserted at evenly-spaced intervals in a packet of stackable potato chips led to higher and more accurate consumption estimates, and a reduction in food intake, relative to ‘unsegmented’ packets of potato chips (Geier, Wansink, & Rozin, 2012). This is a relatively robust finding and not limited to judgements about food (e.g, Pelham, Sumarta, & Myaskovsky, 1994 reported evidence for use of a ‘numerosity heuristic’ in judgements of quantity for non-food items).

However, to date studies have tended to focus on effects of segmentation on *ad libitum* intake and the effect on beliefs about food remains unexplored. In many cases (if not the majority) meals are pre-selected and then consumed in their entirety (Fay, Ferriday, et al., 2011). On this basis, it is argued that meal size is often planned and determined before a meal begins (Brunstrom, 2011). In a number of studies, Brunstrom et al. suggest that ‘expected satiety’ (ES; expected relief from hunger between meals) plays a key role in portion-size selection (Brunstrom and Rogers, 2009, Brunstrom et al., 2011). ES independently predicts self-selected ‘ideal’ portion sizes, both in computerised measures (Brunstrom and Rogers, 2009, Brunstrom and Shakeshaft, 2009) and in actual portion selections (Wilkinson et al., 2012). It is also associated with the amount (kcal) of food consumed in a meal (Wilkinson et al., 2012) and with the satiety experienced after it has terminated (Brunstrom et al., 2011, Fay et al., 2011b). One possibility, therefore, is that segmentation also influences ES.

In the current study, we tested the hypothesis that the ES of a food can be increased by presenting it in multiple small units, and that the extent to which this increase is observed is dependent on the degree of segmentation (number of units) but not on the specific food or the absolute portion size that is presented. To test this proposition, equicaloric portions of different foods were presented in one, three, and six units. ES was assessed using a previously validated ‘method of adjustment’ (see Brunstrom, 2011 for review). Previously, this approach has been used to quantify relative differences in ES across foods. In this specific instance we also considered alternative approaches that provide an indication of the *absolute* effect of segmentation on ES. We selected a novel implementation of magnitude estimation, an approach often used by psychophysicists to quantify absolute intensity and size judgments (Stevens, 1957, Stevens, 1975). This provides a means of calculating a % increase in anticipated relief from hunger that is produced by increasing levels of segmentation. Finally, following other studies (e.g., Brogden & Almiron-Roig, 2010), we also assessed ES using a visual-analogue scale.

A further objective was to determine whether segmentation changes the perceived volume (PV) of a food. Specifically, when presented in multiple smaller units, the physical size of a food may appear larger relative to when it is presented as an (equicaloric) single unit. Previously, measures of PV appeared to explain some of the variation in ES across foods (Brunstrom et al., 2010, Keenan et al., 2015). Therefore, the effect of segmentation on ES might be explained by a change in PV. To explore this idea we quantified the PV of our test foods (using a method of adjustment and magnitude estimation) and used these measures to determine the extent to which effects of segmentation of ES can be explained by changes in PV.

## 2 Method

### 2.1 Overview

Participants evaluated the ES and PV of three test foods; salted peanuts, spaghetti Bolognese and chicken tikka masala (supplementary materials). These foods were selected because they are commonly consumed in the UK. Each food was presented and evaluated in five different portions; 200, 400, 600, 800 and 1000 kcal. Each portion was presented in one of three different levels of ‘segmentation’, (a) a single combined portion (low segmentation), (b) three equal segments (medium segmentation), and (c) six equal segments (high segmentation). In combination, this yielded a total of 45 test stimuli (3 foods x 5 portions x 3 levels of segmentation). All participants evaluated every test stimulus and completed all measures. Participants could pause at any point during each stimulus block to minimise fatigue.

### 2.2 Participants

Sixty-eight participants (20 male and 48 female) were recruited from the undergraduate population at the University of Bristol and from the surrounding area. Vegetarians and vegans were excluded. Participants received either a course credit or £7 (sterling) in return for their participation. Ethical approval was granted by the local Faculty of Science Research Ethics Committee.

### 2.3 Image preparation and test foods

Table 1 contains a summary of the macronutrient composition of the three test foods; two ‘main meals’ (spaghetti Bolognese, tikka masala) and a snack (salted peanuts). All were supplied by Sainsbury's Ltd, UK. Images were captured using a Nikon D50 camera and were presented on a 24-inch widescreen TFT-LCD monitor. Test foods were prepared according to manufacturer instructions and photographed on a square 300 mm by 300 mm plate. Each test food was photographed with three levels of segmentation and in five portion sizes (see supplementary materials), rendering 15 images in total. We selected rice with vegetables (Uncle Ben's Express Golden Vegetable Rice, Knorr) as a comparison food in the method of adjustment task (see ‘expected satiety’ below). Images were taken of 101 portions that spanned the range 10 kcal–1000 kcal with logarithmic spacing. Each portion was presented on a round 255-mm diameter plate.

**Table 1:** Calorie and macronutrient content of the comparison foods (all values typical per 100 g).

	Kcal	Protein (g)	Carbohydrate (g)	Fat (g)	Fibre (g)
Spaghetti Bolognese	162	7.3	16.4	7.1	1.7
Chicken tikka masala	178	8.1	19.5	7.2	1.5
Jumbo salted peanuts	639	29.5	13.3	52	5.8
Rice with vegetables	150	3.1	29.6	2.1	0.7

### 2.4 Measures

The following measures were implemented using custom software written in Microsoft Visual Basic 6.0.

***Appetite ratings.*** Participants rated their hunger and fullness on a 100-mm visual-analogue scale (VAS) anchored by “not at all” and “extremely” on the left and right, respectively.

***Food familiarity.*** Participants were asked to indicate their familiarity with an un-segmented 200-kcal portion of each test food, presented in randomised order. The familiarity task required participants to indicate, using one of 4 drop-down menus (per day; per week; per month; per year), how often they consumed each comparison food. The familiarity scores were converted to a common unit – number of times consumed per year.

***Expected satiety (method of adjustment.)*** Following an earlier study (Brunstrom & Rogers, 2009), in separate trials, participants adjusted the size of a ‘comparison food’ to match the satiety that was expected from each test food (the ‘standard food’). Respectively, the standard and the comparison food were presented on the left- and right-hand side of the screen. Participants responded to the instruction “In this task you will be shown two foods. In this task you should: 1. Look at the food on the left. Imagine you are having this plate of food for lunch today and you won't be eating again until your evening meal; 2. Change the portion of food on the right so that both foods will keep you feeling satisfied (*i.e.*, stave-off hunger) for the same amount of time.” The order of the test foods was randomised across participants and the initial comparison portion was selected randomly in each trial. Participants used the arrow keys on the keyboard to manipulate the size of the comparison food.

**Fig. 1:**
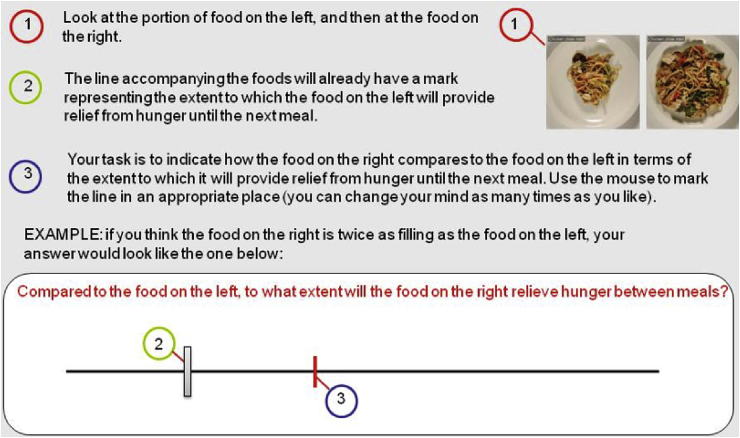
Instructions for the magnitude estimation ES task.

***Expected satiety (magnitude estimation).*** The purpose of the magnitude-estimation measure of ES was to remove the need for participants to manipulate one food to create a match with a different comparison food (as in the method of adjustment task, described above). In this task, the test food was presented on the right-hand side of the screen. On the left-hand side the participants were shown an unsegmented (single unit) 300-kcal portion of the same type of food. Participants were presented with a horizontal scale with a single short vertical line that intersected the horizontal 15 mm from the left. Participants were told that this line represented the extent to which the food on the left would provide relief from hunger until the next meal (Fig. 1). The position of the vertical mark on the line and the amount of the food (standard) shown on the left were chosen arbitrarily, since they simply represented a standard against which all other portions of the same food were compared. Participants were instructed as follows. “Your task is to indicate how the food on the right compares to the food on the left in terms of the extent to which it will provide relief from hunger until the next meal. Use the computer mouse to mark the line in an appropriate place.” At the beginning of the task the participants were shown an example to demonstrate a response indicating that the portion on the right was considered to be twice as filling as the portion on the left. Responses were recorded as the distance (mm) from the left of the scale. As in the Method of Adjustment, all test foods, portion sizes, and levels of segmentation were presented in randomised order across participants.

### 2.5 Expected satiety (visual analogue scale)

Participants were initially instructed to, “Imagine you are having one of these portions of food for lunch and you won't be eating again until your evening meal. Compared to your past experiences with different foods, if 0 was most hungry you've ever felt between meals and 100 was the least hungry you have ever felt: How much will this portion of food stop you from feeling hungry between meals? Please consider the whole rating scale when making your response.” Participants used the mouse to place a mark on the scale (anchored by ‘0’ on the left and ‘100’ on the right), and pressed the enter key to move on to the next judgement. Responses were recorded as distance (mm) from the extreme left of the 100 mm scale.

***Perceived volume*** PV was assessed using both the method of adjustment and magnitude estimation. Apart from the instructions, the tasks were identical to the measures of ES. In the method of adjustment the participants were instructed to “look at the picture on the left” and to “change the portion of food on the right so that both foods have the same physical size.” In the magnitude estimation task they were told “Your task is to indicate how the food on the right compares to the food on the left in terms of its volume/physical size.”

***Questionnaire measures*** To characterise dietary trait characteristics of our sample participants completed the dietary restraint section of the Dutch Eating Behaviour Questionnaire (DEBQ; van Strien, Frijters, Berger, & Defares, 1986) and the dietary disinhibition component of the Three Factor Eating Questionnaire (TFEQ; Stunkard & Messick, 1985). At the end of the session, participants were asked “Did you find these tasks easy to understand?” and “Were the tasks easy to complete?” Response options were binary (yes/no). Finally, beliefs about the study aim (demand awareness) were probed with the following instruction, “Please write in the box below (briefly) what you believe the experiment was about.” Responses that mentioned the presentation of the foods in multiple versus single units, or answers suggesting an awareness of this manipulation, were coded as ‘demand aware’.

### 2.6 Procedure

Participants were tested between 09:00 h and 16:00 h. On arrival they provided written consent and then completed computer-based measures of appetite and familiarity, followed by measures of PV and ES (in counterbalanced order; ES or PV tasks first). Both ES and volume estimation tasks were completed in the same order; method of adjustment first, followed by magnitude estimation. Finally, participants completed the questionnaires and their height and weight was recorded.

### 2.7 Data analysis

It was anticipated that, irrespective of the type of food or its portion size, segmenting a food into multiple small units would promote greater ES. It was also hypothesised that the effect of segmentation on ES might be explained by a change in PV. In the first instance, separate mixed linear models were used to evaluate the three measures of ES (magnitude estimation, method of adjustment and VAS). In each case, ‘segmentation’ (one, three and, six units), ‘food’ (spaghetti Bolognaise, chicken tikka masala, and peanuts), and ‘portion size’ (200, 400, 600, 800 and 1000 kcal) were included as fixed factors, and ‘participant’ was entered as a random factor. Previously it has been shown that ES increases as a food becomes more familiar (Brunstrom, Shakeshaft, & Alexander, 2010). Therefore, we included this measure as a covariate in each model. Demand awareness was observed in 20.6% of our sample. Therefore, we also included this binary outcome as a fixed factor in our model. Because three models were explored, we corrected for the inflated likelihood of Type 1 error by applying a more conservative critical acceptance value (*p* = 0.017). The same analysis strategy was used to explore the two measures of PV (magnitude estimation and method of adjustment) and a critical acceptance value of *p* = 0.025 was applied.

Finally, to establish whether PV might explain the effect of segmentation on ES, separate mixed linear models were conducted on the measures of ES, with the corresponding measure of PV (magnitude estimation and method of adjustment) entered as a covariate. As before, fixed factors were segmentation, food, portion size and demand awareness, participant was entered as a random factor, and food familiarity was included as a covariate. Again, we applied a more stringent critical alpha value (*p* = 0.025). For reasons of brevity, unless indicated otherwise, the reader should assume that all unreported comparisons, main effects, and interaction terms failed to reach or approach statistical significance at our Bonferroni corrected alpha levels.

The full dataset has been made available on the Open Science Framework at https://osf.io/j2xfn/.

## 3 Results

### 3.1 Participant characteristics

Participants had a mean age of 22 years (*SD* = 8.3), a mean BMI of 22.7 kg/m^2^ (*SD* = 3.2), a mean DEBQ-restraint score of 2.4 (*SD* = 0.87), and a mean TFEQ-disinhibition score of 7.4 (*SD* = 3.4). At the beginning of the experiment, mean hunger scores were 29.4 mm (*SD* = 25.3) and fullness scores were 51.5 mm (*SD* = 26.3).

### 3.2 Food familiarity

A repeated-measures ANOVA showed that there was a significant difference in participants’ familiarity with the test foods (*F*(2, 134) = 20.54, *p* < 0.001). Planned comparisons showed that the spaghetti Bolognaise was eaten significantly more frequently (*M* = 34.9 times per year, *SE* = 2.98) than peanuts (*M* = 14.5 times per year, *SE* = 2.89; *t*(67) = 5.02, *p* < 0.001) or chicken tikka masala (*M* = 15.9 times per year, *SE* = 2.36; *t*(67) = 5.8, *p* < 0.001), There was no significant difference in frequency of consumption of the latter two foods (*t*(67) = 0.42, *p* = 0.68).

### 3.3 The effect of segmentation on expected satiety (ES)

**Fig. 2:**
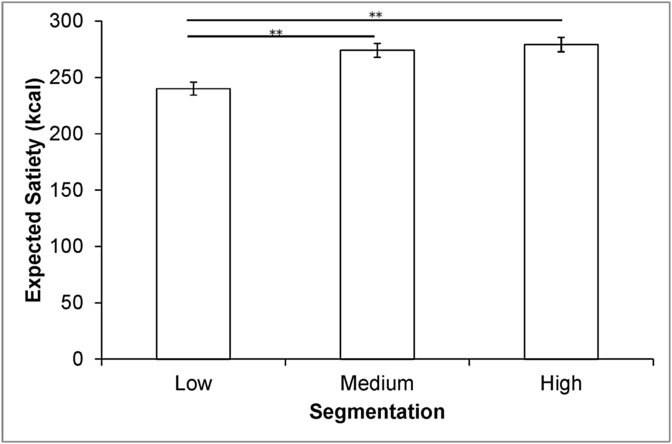
Means and SE for the ES method of adjustment task (data are collapsed across foods and portion sizes; **p < 0.001).

***Method of adjustment*** Consistent with our hypothesis, ES was increased by segmentation (*F*(2,950) = 6.62, *p* = 0.001). Foods segmented into six units were expected to deliver 16% more satiety than foods in a single unit. Pairwise comparisons (Bonferroni) showed that when foods were presented in a single unit they were expected to deliver significantly less satiety than when segmented into three units (*p* < 0.001) or six units (*p* < 0.001). Foods in three and six units did not differ significantly (*p* > 0.05). For associated means (±SE) see Fig. 2.

**Fig. 3:**
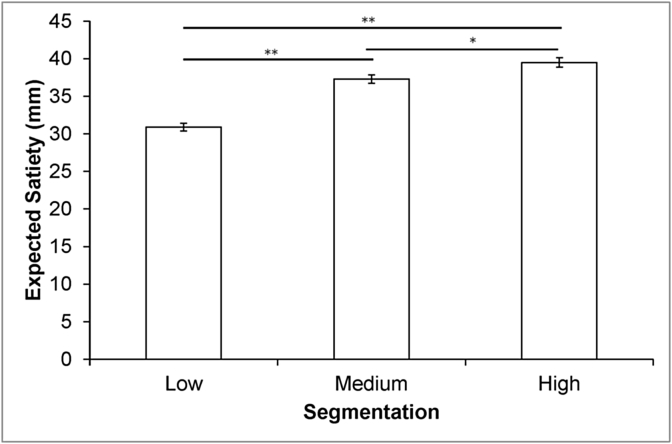
Means and SE for the ES magnitude estimation task (data are collapsed across foods and portion sizes; *p = 0.03; **p < 0.001).

***Magnitude estimation*** Our analysis revealed a significant main effect of segmentation on expected satiety (*F*(2, 1219) = 40.9, *p* < 0.001). Pairwise comparisons (Bonferroni) showed that foods in a single unit were expected to deliver significantly less satiety than the same foods in three (*p* < 0.001) or six units (*p* < 0.001), and that foods in three and six units also differed significantly (*p* = 0.03). In this case, relative to the single-unit format, segmenting the foods into six units generated a 28% increase in ES. For associated means (±SE) see Fig. 3.

***VAS.*** There was no significant effect of segmentation on ES (*F*(2,1556) = 0.692, *p* = 0.501).

### 3.4 The impact of segmentation on PV (PV)

**Fig. 4:**
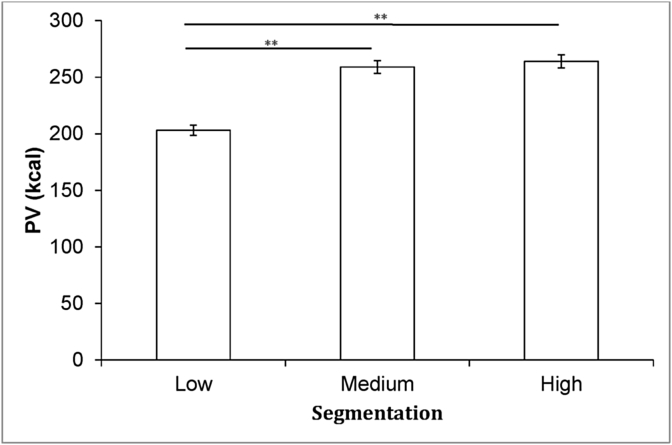
Means and SE for the PV method of adjustment task (data are collapsed across foods and portion sizes; **p < 0.001).

***Method of adjustment*** There was a significant effect of segmentation on PV (*F*(2,715) = 21.7, *p* < 0.001). *Post-hoc* pairwise Bonferroni-corrected comparisons showed that the PV of single-unit foods was significantly smaller than foods presented in three (*p* < 0.001) and six units (*p* < 0.001). The difference between three and six-units failed to reach significance (*p* > 0.05). Fig. 4 shows that segmenting single-unit foods into six units increased their PV by 30%.

***Magnitude estimation*** There was a significant main effect of segmentation on PV (*F*(2,1149) = 73.8, *p* < 0.001). Pairwise comparisons (Bonferroni) showed that the PV of foods in a single unit (*M* = 30.9 kcal, *SE* = 0.48) was significantly smaller than foods in three (*M* = 37.8 kcal, *SE* = 0.48; *p* < 0.001) and six units (*M* = 42.5 kcal, *SE* = 0.62; *p* < 0.001), and that foods in three units were perceived to be significantly smaller than foods in six units (*p* < 0.001).

**Fig. 5:**
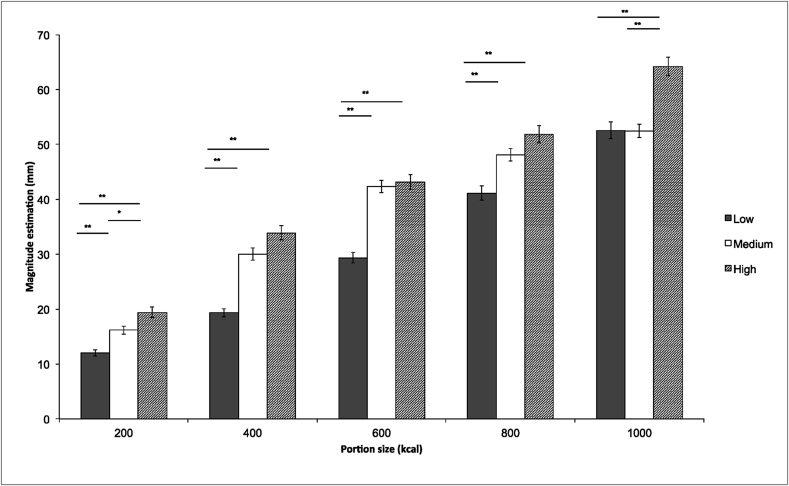
Means and SEs for the PV (PV) magnitude estimation task (*p < 0.05; **p < 0.001) by portion size (kcal) and level of segmentation (low, medium, high).

However, the segmentation effect was not consistent across portion sizes (significant portion size*segmentation interaction, *F*(8, 475) = 3.1, *p* = 0.002). Briefly, pairwise comparisons (Bonferoni) showed that the effect of specific levels of segmentation appeared to vary with portion size, but not systematically, suggesting that the interaction may be spurious; specifically, a segmentation effect was evident at all levels when foods were presented in 200-kcal portions, at all levels except between medium and high segmentation when foods were presented in 400, 600 and 800 kcal portion sizes and at all levels except between low and medium when foods were presented in a 1000 kcal portion size. Fig. 5 shows mean (SE) PV across segmentation conditions and portion sizes.

### 3.5 Impact of segmentation on ES when controlling for PV

***Magnitude estimation*** When entered into our model, PV was a significant covariate (*F*(1,749) = 11.1, *p* = 0.001) and the previously significant main effect of segmentation on expected satiety failed to achieve significance (*F*(2,1020) = 2.9, *p* = 0.055).

***Method of adjustment*** When entered into our model, PV was a significant covariate (*F*(1,1706) = 107, *p* < 0.001) and the main effect of segmentation on expected satiety was no longer significant (*F*(2,813) = 0.535, *p* = 0.586).

### 3.6 Hunger and fullness

Reanalysis of the above data with hunger and fullness included as covariates in level 1 of the mixed linear model revealed no significant interactions between hunger and fullness and segmentation.

### 3.7 Demand awareness

The only significant effect involving demand awareness was a demand awareness by segmentation interaction for the PV magnitude estimation task. The nature of this interaction is shown in the Supplementary Materials.

## 4 Discussion

We observed a clear and consistent main effect of segmentation on ES. In the magnitude estimation task, this amounted to a 28% increase in ES when the foods were presented in six units relative to a single unit. Previously, Chang et al. (2012) found that participants consumed less rice when it was presented in multiple small units (triangles or balls) compared to a single unit. Our findings extend this work by showing that segmentation impacts beliefs about a meal before it is consumed. This effect of segmentation was present across foods with different energy densities and different types of foods (e.g. meal and snack foods). Previously, we have shown that ES is a strong predictor of food intake and subsequent satiety (Wilkinson et al., 2012). Therefore, one possibility is that the effect of segmentation on ES played a causal role in mediating previous observations (Chang et al., 2012, Marchiori et al., 2012, Marchiori et al., 2011, Wadhera et al., 2012, Weijzen et al., 2008).

However, we failed to find a main effect of segmentation on ES using a VAS task (effects were only observed using our ‘method of adjustment’ and ‘magnitude estimation’ tasks). One possibility is that in the context of this study our VAS measure lacked sensitivity and therefore segmentation effects were not detected. Unlike the VAS measure, the ‘method of adjustment’ and ‘magnitude estimation’ tasks are forms of psychophysical techniques, often used by researchers of sensory perception. Importantly, such methods are highly sensitive (see Brunstrom, Shakeshaft, & Scott-Samuel, 2008 for discussion of the use of psychophysics to measure expected satiety).

We also observed a significant effect of segmentation on PV. Indeed, using the method of adjustment task, the effects of segmentation on volume-estimation and ES were very similar. Using the magnitude estimation task, the effect of segmentation on PV varied across portion sizes (i.e., significant portion size*segmentation interaction) whereas for ES the effect was consistent across foods and portion-sizes. Nonetheless, for both tasks, the effect of segmentation on ES was no longer significant after controlling for PV, indicating that the effect of segmentation is likely to be governed by a change in PV. In other words, when presented in multiple smaller units, foods appeared larger and they were evaluated as having relatively higher ES for this reason. Although this explanation remains to be tested formally (an explanation around reverse causality cannot be ruled out here due to the design of this study), it may be relevant that evidence for segmentation has also been observed in rodents (Capaldi et al., 1989, Wadhera et al., 2012), which is consistent with a mechanism involving relatively low-level processing.

An alternative explanation is that the effect of segmentation on ES reflects a ‘standard unit bias.’ Geier and Rozin (2009) have shown that participants overestimate calories in smaller-than-normal portions and interpret this as a form of estimation bias. One possibility is that segmenting a portion into separate smaller units generates a similar bias. This possibility might be explored by asking participants to estimate the number of calories in food portions where the level of segmentation is systematically varied.

A third possibility is that the segmentation effect was due to the anticipation of sensory-specific satiety (the decline in pleasantness of a food as it is eaten relative to 'uneaten' foods; SSS; Rolls, Rolls, Rowe, & Sweeney, 1981). Previously, Weijzen et al. (2008) demonstrated that nibble-sized chocolate-covered wafer snacks are consumed in smaller amounts when compared with an otherwise identical single whole wafer. Weijzen et al. (2008) suggested that the smaller bars were eaten at a slower rate and, in turn, this increased oral exposure and earlier onset of SSS. More recently, Wilkinson, Hinton, Fay, Rogers, and Brunstrom (2013) have shown that the variety effect (thought to be underpinned by sensory specific satiety; Rolls, 1986) is anticipated during meal planning. Therefore, one possibility is that when shown a highly segmented test food, our participants anticipated greater SSS, and reported higher ES on this basis.

The current study provides novel insight into the effect of segmentation on ES. However, with regard to the broader effect on *ad libitum* food intake, an alternative explanation is that segmentation influences perceptions of portion-size appropriateness and impulsiveness. In previous research, it was found that consuming five small units of chocolate was considered to be more impulsive and less appropriate than consuming the same amount of chocolate as one single unit (Van Kleef, Kavvouris, & van Trijp, 2014). Furthermore, in these studies, the effect of smaller versus larger units on subsequent intake of chocolate was mediated by perceived impulsiveness (Van Kleef et al., 2014). These different accounts (i.e., ES vs. perceived impulsiveness) should be scrutinized in future studies.

In the current study, the foods were presented and evaluated in a computer-based task and were not presented in three dimensions. The impact of this procedure remains to be determined, although assessments of this kind appear to be a good predictor of physical food portion selections and also subsequent intake at a meal (Wilkinson et al., 2012). Despite the persuasive evidence that segmentation influences food intake and perceptions of amount consumed, many meals are not consumed *ad libitum*, but are instead pre-selected and then consumed in their entirety. Since ES is a strong predictor of potion size choice and later food intake, future studies should seek to confirm the anticipated impact of segmentation on portion size choices and later intake. In addition, the present study assessed the impact of segmentation on ES and perceived volume for amorphous foods only. A useful future study could be to compare these effects in ‘non-amorphous’ or unit foods, such as sandwiches or other ‘picnic-type’ foods. Finally, an opportunity now exists to capitalise on the phenomenon demonstrated here. Specifically, commercial food manufacturers might consider presenting smaller-size units to increase ES. This might be especially effective in products that are designed to confer benefits for healthy weight maintenance.

## Funding

RO-C was supported by a BBSRC-DRINC PhD studentship (grant agreement no. BB/G005443/1) at the time the research was undertaken, and the research is also linked with a BBSRC-DRINC project on reducing portion size (grant agreement no. BB/L02554X/1). Charlotte A. Hardman receives research funding from the American Beverage Association and personal fees from the International Sweeteners Association.
