# Supplementary material for: Presenting a food in multiple smaller units increases expected satiety
Source: Appetite. 2017 Nov 1;118:106–12. doi: 10.1016/j.appet.2017.07.024 (PMC5590487; doi:10.1016/j.appet.2017.07.024)
Supplement: Online data [file mmc1.docx]

**Supplementary materials**

***S1. Example test foods***

*
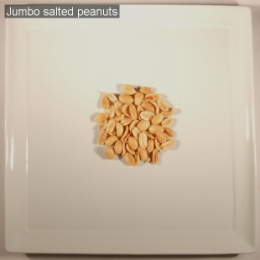

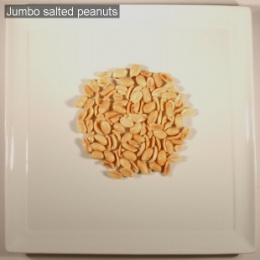

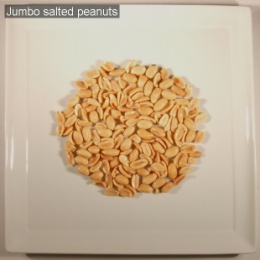

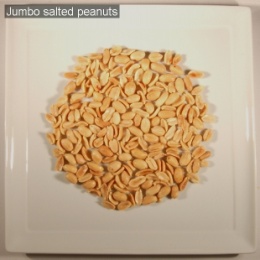

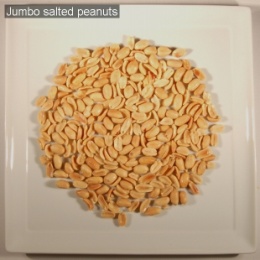
*

Low

*
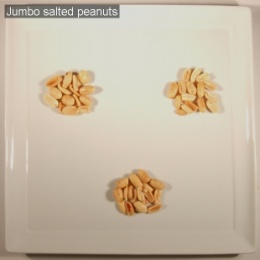

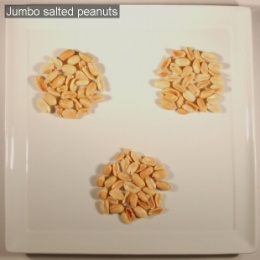

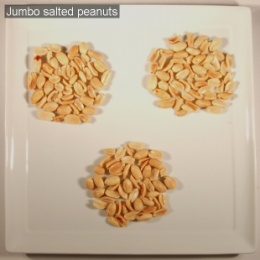

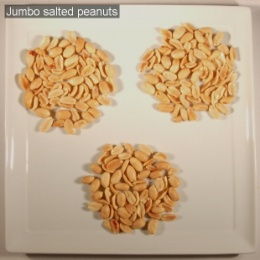

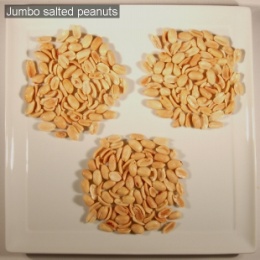
*

Medium

*
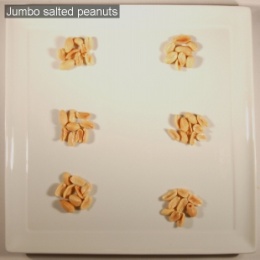

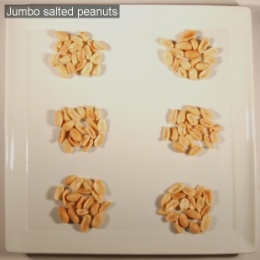

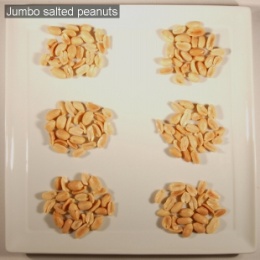

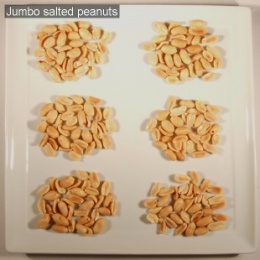

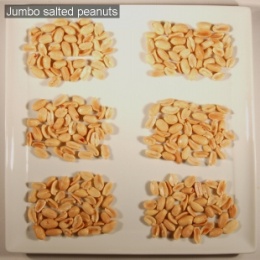
*

High

*Figure S1 a.* Jumbo salted peanuts displayed in 3 levels of segmentation (low, 1 unit; medium, 3 units; high, 6 units) and 5 portion sizes (L-R: 200, 400, 600, 800 and 1000 kcal).


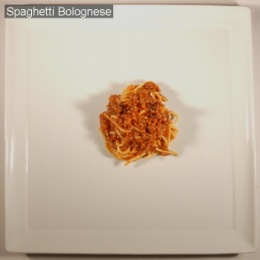

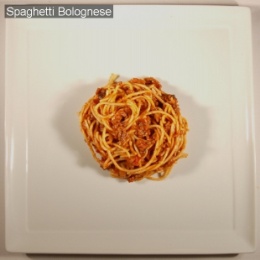

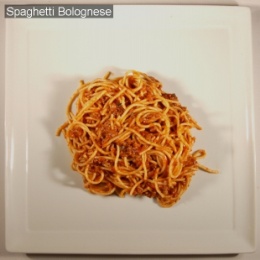

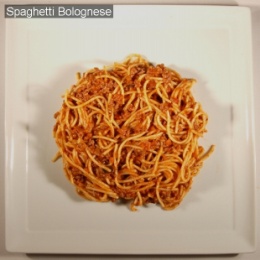

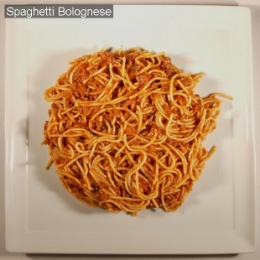


Low


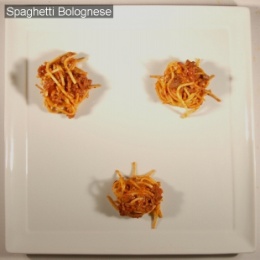

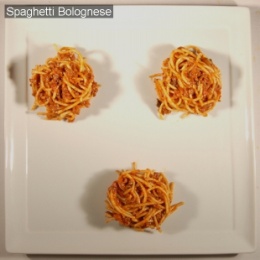

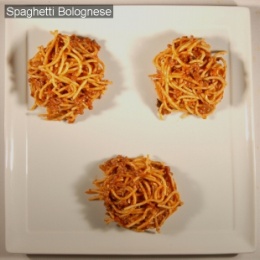

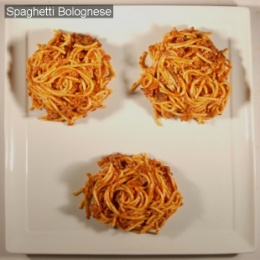

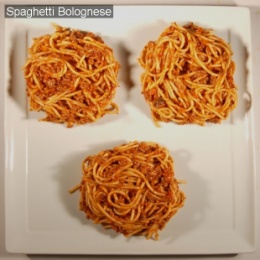


Medium


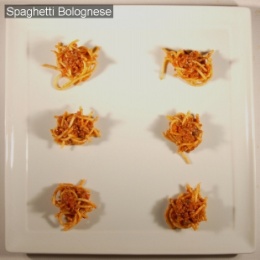

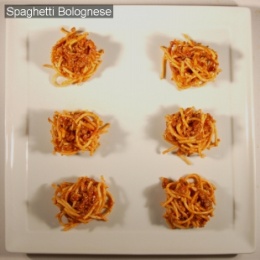

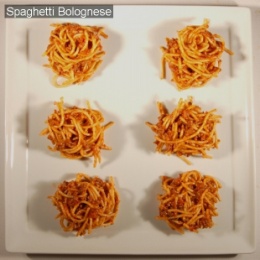

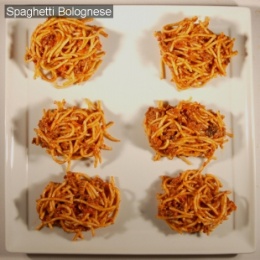

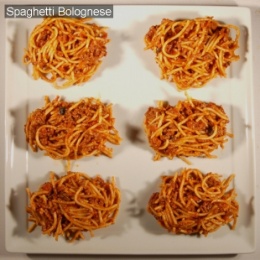


High

*Figure S1 b.* Spaghetti Bolognese displayed in 3 levels of segmentation (low, 1 unit; medium, 3 units; high, 6 units) and 5 portion sizes(L-R: 200, 400, 600, 800 and 1000 kcal).


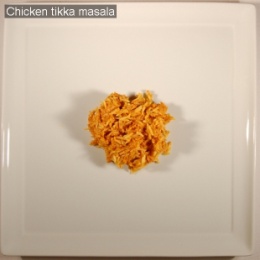

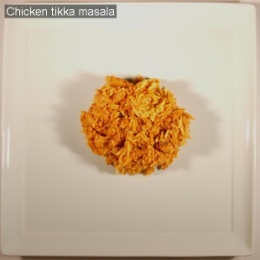

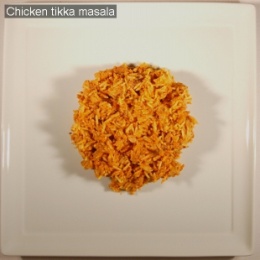

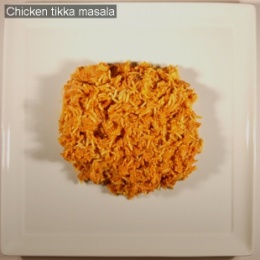

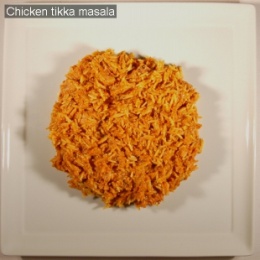


Low


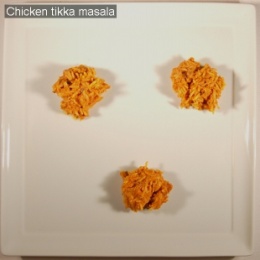

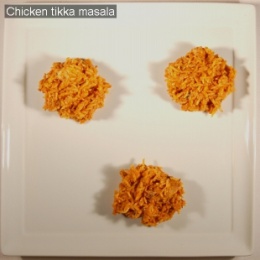

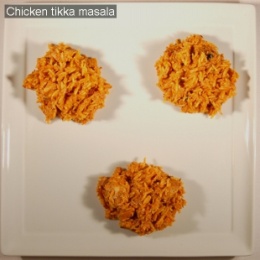

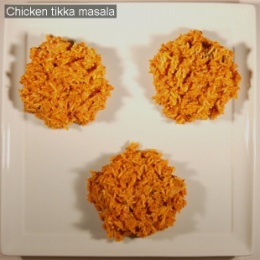

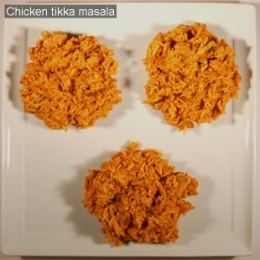


Medium


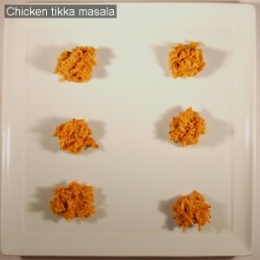

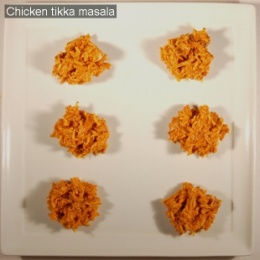

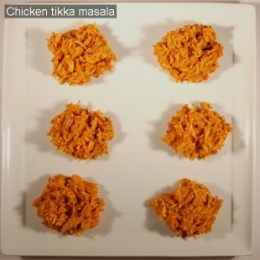

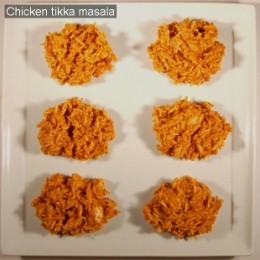

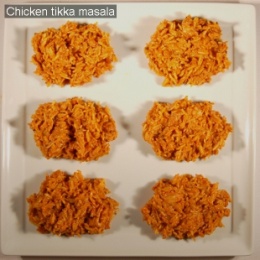


High

*Figure S1 c.* Chicken tikka masala displayed in 3 levels of segmentation (low, 1 unit; medium, 3 units; high, 6 units) and 5 portion sizes (L-R: 200, 400, 600, 800 and 1000 kcal).

***S2. Demand awareness***

There was a significant demand awareness* segmentation interaction in perceived volume (PV) for the magnitude estimation task (*F*(2, 1149) = 4.6, *p* = .01). Pairwise comparisons showed that an effect of segmentation was evident at every level for both demand aware and unaware participants. Generally, PV for foods was higher in the unaware participant group except for at high segmentation level when this trend was reversed. Figure S2 shows mean and SE perceived volume across segmentation and demand awareness groups. All other segmentation interaction effects were not significant (*p* > .025).

There were no other significant demand awareness*segmentation interactions.

*Figure S1.* Mean and SE for the PV magnitude estimation task (***p* < .001)
